# A Comprehensive Investigation of Steroidogenic Signaling in Classical and New Experimental Cell Models of Adrenocortical Carcinoma

**DOI:** 10.3390/cells11091439

**Published:** 2022-04-24

**Authors:** Sandra Sigala, Christina Bothou, David Penton, Andrea Abate, Mirko Peitzsch, Deborah Cosentini, Guido A. M. Tiberio, Stefan R. Bornstein, Alfredo Berruti, Constanze Hantel

**Affiliations:** 1 Section of Pharmacology, Department of Molecular and Translational Medicine, University of Brescia, 25124 Brescia, Italy; sandra.sigala@unibs.it (S.S.); a.abate005@unibs.it (A.A.); 2 Department of Endocrinology, Diabetology and Clinical Nutrition, University Hospital Zurich (USZ) and University of Zurich (UZH), 8091 Zürich, Switzerland; christina.bothou@usz.ch (C.B.); stefan.bornstein@uniklinikum-dresden.de (S.R.B.); 3 Electrophysiology Facility (e-phac), Department of Molecular Life Sciences, University of Zurich (UZH), 8057 Zürich, Switzerland; david.pentonribas@uzh.ch; 4 Medizinische Klinik und Poliklinik III, University Hospital Carl Gustav Carus Dresden, 01307 Dresden, Germany; mirko.peitzsch@uniklinikum-dresden.de; 5 Medical Oncology Unit, Department of Medical and Surgical Specialties, Radiological Sciences, and Public Health, University of Brescia at ASST Spedali Civili di Brescia, 25124 Brescia, Italy; deborah.cosentini@gmail.com (D.C.); alfredo.berruti@unibs.it (A.B.); 6 Surgical Clinic, Department of Clinical and Experimental Sciences, University of Brescia at ASST Spedali Civili di Brescia, 25124 Brescia, Italy; guido.tiberio@unibs.it; 7 Diabetes and Nutritional Sciences, King’s College London, London WC2R 2LS, UK; 8 Center for Regenerative Therapies, Technische Universität Dresden, 01307 Dresden, Germany; 9 Paul-Langerhans-Institute Dresden, Helmholtz Center Munich, University Hospital Carl Gustav Carus, Faculty of Medicine, Technische Universität Dresden, 01307 Dresden, Germany; 10 Lee Kong Chian School of Medicine, Nanyang Technological University, Singapore 636921, Singapore

**Keywords:** adrenocortical carcinoma cell lines, steroidogenesis, electrophysiology, genotype, NCI-H295, MUC-1, TVBF-7

## Abstract

Adrenocortical carcinoma is a heterogeneous and aggressive cancer that originates from steroidogenic cells within the adrenal cortex. In this study, we have assessed for the preclinical gold standard NCI-H295 in direct comparison with the more recently established MUC-1 and a here newly reported ACC cell line (TVBF-7) the mutational status of important driver genes (TP53, MEN1, PRKAR1A, CTNNB1, APC, ZNRF-3, IGF-2, EGFR, RB1, BRCA1, BRCA2, RET, GNAS and PTEN), Wnt-signaling specificities (CTNNB1 mutation vs. APC mutation vs. wildtype), steroidogenic-(CYP11A1, CYP17A1, HSD3B2, HSD17B4, CYP21A2, CYP11B1, CYP11B2, MC2R, AT1R) and nuclear-receptor-signaling (AR, ER, GCR), varying electrophysiological potentials as well as highly individual hormone secretion profiles (Cortisol, Aldosterone, DHEA, DHEAS, Testosterone, 17-OH Progesterone, among others) which were investigated under basal and stimulated conditions (ACTH, AngII, FSK). Our findings reveal important genetic and pathophysiological characteristics for these three cell lines and reveal the importance of such cell-line panels reflecting differential endocrine functionalities to thereby better reflect clinically well-known ACC patient heterogeneities in preclinical studies.

## 1. Introduction

**Figure 1:**
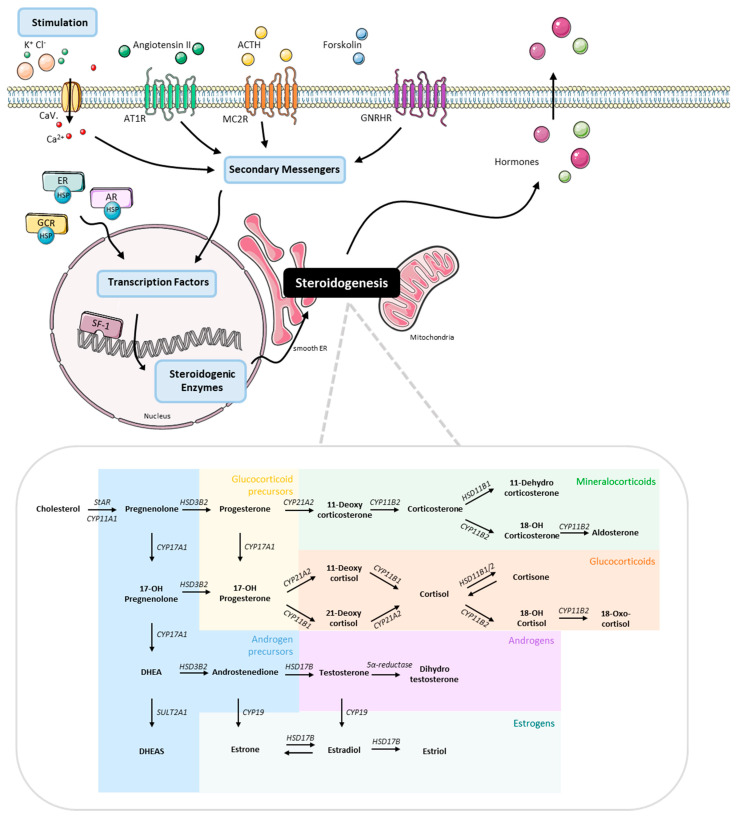
Schematic representation of steroidogenesis pathway from stimulation to hormone production. Figures have been created modifying an image set from Servier Medical Art (SMART) http://smart.servier.com/ (accessed on 19 July 2021).

Adrenocortical carcinoma (ACC) is a rare and aggressive cancer that originates from steroidogenic cells within the adrenal cortex with an estimated incidence of 0.7–1 cases per million of the population per year [1]. ACCs demonstrate heterogeneous steroid secretion patterns producing hormones such as mineralocorticoids, glucocorticoids and androgens or secrete precursor metabolites, deriving from intermediate steps along the three major adrenocortical biosynthetic pathways ([2], Figure 1). Only in rare cases ACCs are biochemically inactive and the elucidation of the steroid profile of ACCs is important in diagnostic, but also in therapeutic level.

In recent years, steroid profiling of patient plasma or 24-h urine samples have been developed and provide steroid metabolomic information via liquid chromatography tandem mass spectrometry (LC–MS/MS) or gas chromatography mass spectrometry (GC-MS) [3,4]. Those tools combined with machine-learning based approaches have resulted in the identification of distinct malignant steroid “fingerprints” for ACC, which could differentiate benign from malignant adrenal tumor [2,3], but also be used as biomarkers and as a screening tool for early identification of tumor recurrence [5]. On the other hand, steroidogenesis is the direct or indirect target of action of drugs used as chemotherapeutics in patients diagnosed with ACC, with main representative the gold standard medication: mitotane [6,7].

To date preliminary data obtained for mineralocorticoids and glucocorticoids show that the steroidogenic pattern could be related with the disease outcome [8,9,10]. However, detailed mechanistic insights are lacking and as indicated above, also androgens and androgen receptor signalling is commonly present in ACC. The androgen receptor (AR) is a ligand-activated transcription factor that plays an important role in the context of various severe diseases. The AR is already a therapeutic target for prostate cancer and is also emerging as a new marker and potential therapeutic target for breast cancer. The availability of selective AR inhibitors (e.g., bicalutamide, enzalutamide, apalutamide) approved for the treatment of prostate cancer might have, thus, potential to be translated to other endocrine cancers. However, for breast and prostate cancer AR-signalling appears to have different functions according to the specific subtype [11], and disease stage [12]. Steroid hormones other than the classical ligands testosterone and dihydrotestosterone may be in this context also of interest considering their potential role in the development of resistance mechanisms [12].

However, important pre-requisite for detailed mechanistic and therapeutic studies are preclinical models. Considering that primary cell cultures from adrenal tumors are, due to rarity of this tumor type, overall limited, many groups have attempted to establish cell lines from ACCs and currently different ACC cell lines are meanwhile available [13,14,15,16,17]. The most implemented model for the study of steroidogenic gene expression and chemotherapeutic responsiveness in ACC until today is NCI-H295, which was established in the early 1990s from Gazdar et al. from a female patient diagnosed with a primary ACC [14]. Extensive in vitro studies have shown that the original line NCI-H295 expresses in baseline all the enzymes participating in normal human adrenal steroidogenic gene expression, including the enzymes that catalyse the rate- limiting steroidogenic step (STAR and CYP11A1) and all the major biosynthetic steroidogenic enzymes (CYP17, HSD3B2, CYP21, CYP11B2, CYP11B1 and HSD17B4) and retain, thus, the general potential to produce all major adrenal steroids. Considering that adrenocortical cells are, normally, excitable cells, the precise control of the membrane voltage is very important for the initiation of steroid synthesis. However, in context of steroidogenesis it should be also mentioned that clinical ACC cases of combined mineralocorticoid and glucocorticoid secretion, as reflected by NCI-H295, are comparably rare. Thus, from a clinical point of view, these cells do maybe not represent the most common phenotype in terms of ACC functionality [18]. Likewise, combined mutations in the two ACC driver genes TP53 and CTNNB1, as described for NCI-H295, is for ACC clinically rather rarely observed [19]. However, due to a lack of ACC models for many years, comprehensive data from additional human models reflecting diverse subtypes are nowadays for many aspects still lacking.

A recently developed, highly emerging model in this field is MUC-1, which has been established as patient-derived tissue xenograft and cell line from an ACC neck metastasis of a male patient [15]. MUC-1 was previously presented to be SF-1 and 3βHSD positive and mice bearing MUC-1 xenografts had increased plasma cortisol [15]. Meanwhile, MUC-1 has been implemented in a variety of preclinical ACC studies and demonstrated repeatedly a different response pattern and clinically frequently observed drug resistance phenotype compared to NCI-H295R [20,21,22,23,24,25,26,27,28,29].

Here, we describe the development and characterization of a new ACC cell line, named TVBF-7, which is carrying a nonsense APC and represents functional signs of autonomous cortisol secretion. In a comprehensive study, we investigate TVBF-7in direct comparison to NCI-H295 and MUC-1 regarding mutational status of main driver genes, steroidogenic signaling, electrophysiological properties as well as secretion profiles and reveal patterns of genetical, steroidogenic distinct and highly relevant pathophysiological ACC sub-types.

## 2. Materials and Methods

### 2.1. Cancer Cell Lines

NCI-H295R cells were originally obtained from ATCC while MUC-1 cells were previously established by our group [15]. Both cell lines were authenticated again in December 2020 by Microsynth (Balgach, Switzerland) and maintained as previously described [15]. Additionally, the novel cell line described as TVBF-7 was authenticated for multiple passages at the universities Zurich and Brescia (Balgach, Switzerland, and BMR Genomics, Padova, Italy). The cells were cultured in advanced D-MEM-F12 (#12634010, Gibco, Waltham, MS, USA), 10% FBS (#10082147, Gibco), 1% penicillin-streptomycin (#15070063, Gibco), 1% amphotericin B (#15290026, Gibco) and 2 mM glutamine (#A2916801, Gibco).

### 2.2. Whole Genome Sequencing and Genomic Data Presentation

**Figure 2:**
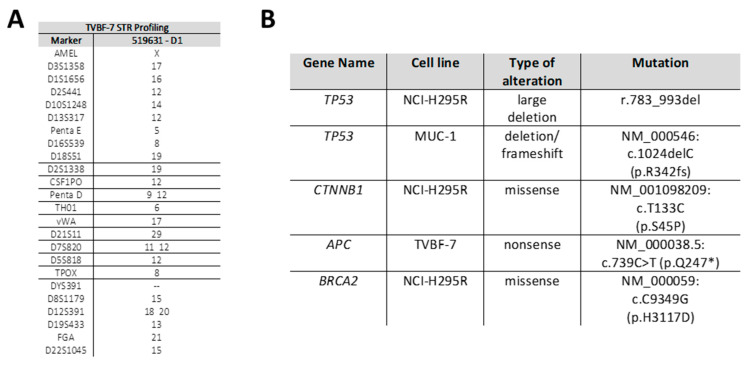
STR profiling of the newly presented TVBF-7 cells (**A**). Most important pathogenic mutations of cancer driver genes as detected by the whole genome sequencing of all three cell lines (**B**).

Whole genome sequencing (WGS) for NCI-H295R, MUC-1 and TVBF-7 cells has been performed and standard bioinformatic analysis has been carried out (BGI, Shenzhen, Guangdong, China). The filtered reads were aligned to the human reference genome (UCSC build HG19) using Burrows-Wheeler Aligner (BWA) software. Single Nucleotide Polymorphisms (SNP), insertions and deletions (InDel) and copy number variations (CNVs) have been annotated (SnpEff tool: http://snpeff.sourceforge.net/SnpEff_manual.html (accessed on 19 July 2021) and VEP tool: https://asia.ensembl.org/info/docs/tools/vep/index.html (accessed on 19 July 2021)) and filtered as presented in detail in the Supplementary File. The data were delivered post-analysis in vcf files, which were further assessed by the use of EmEditor (Washington, DC, USA) for the generation of the information provided in Figure 2B and in the Supplementary Table S3.

### 2.3. Stimulation Experiments

On the treatment starting day, the cell medium was removed, and the cells were washed with PBS and then treated with the following agents: potassium chloride (KCl) (5, 10, 20 mM concentrations added on top to the baseline K^+^ concentration contained in the medium calculated to around 5 mM), (#P5405, Sigma-Aldrich, Buchs, Switzerland), AngII (20, 100, 250 nM) (#A9525, Sigma-Aldrich), ACTH (5, 15, 25 nM) (#A0423, Sigma-Aldrich) or FSK (5, 10, 25 μM) (#F3918, Sigma-Aldrich). Each concentration of the samples and each control was included in triplicate. The plates were incubated at 37 °C and 5% CO_2_ for 24 h, and then either processed for quantitative Real-Time PCR in 24-well plates (TPP #92024; NCI-H295R (200,000/well), MUC-1 cells (85,000/well), TVBF-7 (180,000/well)) or for steroid measurements in the cell supernatant with simultaneous protein quantification within the cells in 6-well plates (TPP#92006; NCI-H295R (1,000,000/well), MUC-1 (425,000/well) and TVBF-7 (750,000/well)).

### 2.4. Quantitative Real-Time PCR

After 24h of stimulation, cells were prepared for total RNA isolation, genomic DNA removal and cDNA generation as previously described [20]. For real-time PCR analysis, EvaGreen^®^ reaction mix (#1725200, Bio-Rad, Hercules, CA, USA) in QuantStudio5 (applied biosystems, Waltham, MS, USA) was used. The primers used are described in Supplementary Table S2. Differences in the threshold cycle (Ct) values between the GAPDH housekeeping gene and the gene of interest (ΔCt) were then calculated as an indicator of difference in the amount of mRNA expressed, corrected for the efficiency of the reaction previously acquired via standard curve.

### 2.5. Liquid Chromatography Tandem Mass Spectrometry (LC–MS/MS) Steroid Measurements

After 24h of stimulation, cell supernatant was collected, directly frozen on dry ice and stored at. −80 °C. A whole panel of steroid metabolomics was determined by LC–MS/MS as described previously (Peitzsch et al., 2015). The results were provided in concentration (ng/mL). As blank the respective medium supernatant for similarly treated but cell free wells was used for untreated and stimulated conditions. For this experiment, the following concentrations have been used: KCl 10 mM, AngII 100 nM, FSK 10 μM and a low (l) and a high ACTH (h) concentration corresponding to 10 and 50 nM, respectively.

### 2.6. Protein Quantification

Protein samples from the wells used for supernatant collection were acquired, following the same order. More specifically, cell proteins were extracted in RIPA buffer (50 mM Tris pH 8.0, 150 mM NaCl, 0.01 *v*/*v* NP-40 #74385, Sigma-Aldrich, St. Louis, MI, USA), 0.005 *v*/*v* sodium deoxycholate (#D6750, Sigma-Aldrich), and 0.001 *w*/*v* SDS (#2326.3, Roth, Karlsruhe, Germany) supplemented with a complete protease inhibitor cocktail (#11836170001, Roche, NY, USA) and phosphatase inhibitor cocktail (#P5726, Sigma-Aldrich). The homogenized lysate was centrifuged at 16,000× *g* for 15 min and protein concentration was quantified by Pierce BCA Protein Assay (#23225, Thermo Scientific^TM^, Reinach, Switzerland) following the manufacturer’s recommendations.

### 2.7. Electrophysiological Studies

For whole-cell automated patch clamp, NCI-H295R, MUC-1 and TVBF-7 cells were grown until ~80% confluency. Cells were used immediately after detaching with Accutase (Sigma-Aldrich, Buchs, Switzerland) and resuspended in an extracellular solution containing (in mM) 135 NaCl, 1.8 MgCl_2_, 1.8 CaCl_2_, 10 HEPES, 5 KCl, pH 7.4 adjusted NaOH/HCl. 3.0 × 10^6^ cells/mL and then directly added to the centrifuge tube of the QPatchII automated patch clamp platform (Sophion Bioscience, Ballerup, Denmark). 48X single hole QChips with a resistance of ~2 MΩ were used for experiments with an intracellular solution containing (in mM) 95 K-gluconate, 30 KCl, 4.8 Na_2_HPO_4_, 1.2 NaH_2_PO_4_, 5 glucose, 2.38 MgCl_2_, 0.73 CaCl_2_, 1 EGTA, 3 Mg-ATP, pH 7.2 adjusted KOH/HCl. No seal enhancer and no liquid junction potential correction (11 mV measured for the solution pair) was used. Only cells maintaining a seal resistance of >0.5 GΩ, and with stable access resistance throughout the experiment, were used. After obtaining the whole-cell patch clamp, the extracellular solution was exchanged twice with solution containing 5, 15, 50 mM K^+^ and a protocol including 20 mv voltage steps (−100 to +80 mV, holding potential −60 mV, 200 ms) was applied every 50 s. The osmolality of the extracellular solution was kept constant by proportionally decreasing the concentration of NaCl for increasing concentrations of KCl (e.g., in mM 135 NaCl + 5 KCl or 90 NaCl + 50 KCl). Current density was calculated using steady state current (average of last 100 ms of each step) divided by the cell capacitance measured immediately before each protocol. For the effect of AngII (20 nM), FSK (5 μM) and ACTH (5 nM), cells were washed twice with extracellular solution containing 5 mM K^+^ before the application of the effector. Voltage protocols including 20 mv voltage steps (−100 to +80 mV, holding potential −60 mV, 200 ms) was applied. For each treatment, currents and reversal potential were measured.

### 2.8. Statistical Analysis and Graphical Designs

Statistical analysis and graphical representation of the data was carried out using GraphPad Prism software (version 8, GraphPad Software, La Jolla, CA, USA). If not stated otherwise, comparison between control group and two or more treatment groups or between cell lines (mean of each) were performed by one-way ANOVA followed by Dunnett’s multiple comparisons test. The data are presented in column graphs depicting the mean ± SEM. The statistical significance is denoted as stars in the graphs (* *p* < 0.05; ** *p* < 0.01; *** *p* < 0.001).

For the representation of the graphical abstract of Figure 1, the figures created by modifying an image set from Servier Medical Art (SMART) http://smart.servier.com/ (accessed on 19 July 2021), which is cited appropriately.

## 3. Results

### 3.1. Establishment of a Novel Cancer Cell Line, TVBF-7

Recently, ACC primary cells (primary culture ACC115m [27]) have been isolated from a lymph node metastasis from a male patient without clinically obvious signs of steroid excess, while no functional testing has been performed. When the cells were found to be continuously passageable, the cells were further cultured for multiple passages at the university of Brescia (to date P 34) and remained stable as confirmed by cell authentication performed via STR profiling in passages 7, 14, 16, 23, and 29 (Supplementary Table S1). After transfer to the University of Zurich, the cells were cultured during the subsequently described experiments and again authenticated by STR-Analysis (Figure 2A). The newly established and characterized ACC cell line has been named TVBF-7.

### 3.2. Mutational Status of Important Driver Genes 

The mutational status of TVBF-7 in main driver genes (TP53, MEN1, PRKAR1A, CTNNB1, APC, ZNRF-3, IGF-2, EGFR, RB1, BRCA1, BRCA2, RET, GNAS and PTEN) has been assessed vs. NCI-H295R and MUC-1. In Figure 2B, the most critical mutations are presented, a full panel of findings is provided in the Supplementary Materials. The main differences were detected regarding Wnt-signaling pathway regulators, as our analyses revealed for TVBF-7 a nonsense mutation in APC (Figure 2B). For NCI-H295R, the known CTNNB1 mutation was confirmed while MUC-1 represented the wild type for both genes. Moreover, the analysis revealed TP53 WT for TVBF-7 compared to the TP53 mutated genotypes of NCI-H295R and MUC-1. For BRCA2 a missense mutation was found for NCI-H295R exclusively.

### 3.3. Baseline Gene Expression Levels and Electrophysiological Properties

**Figure 3:**
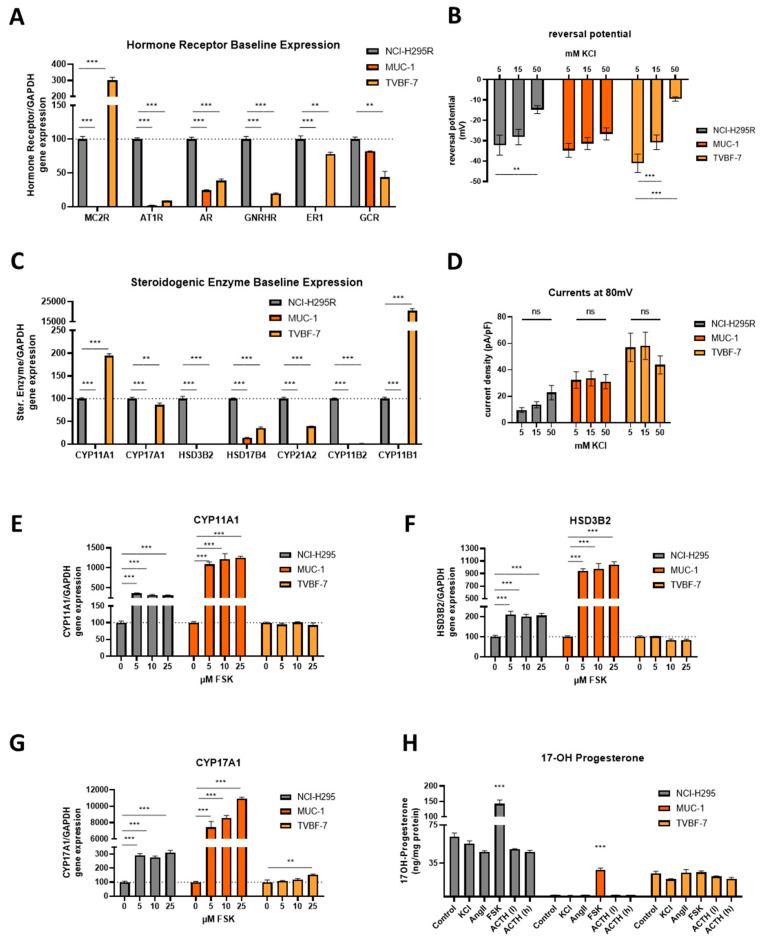
Baseline gene expression levels of different hormone receptors (MC2R, AT1R, AR, GNRH, ER1 and GCR) of unstimulated cells (**A**). Baseline gene expression levels of steroidogenic enzymes (CYP11A1, CYP17A1, HSD3B2, HSD17B4, CYP21A2, CYP11B2 and CYP11B1) (**B**). Comparative depiction of the reversal potential in baseline (5mM KCl) and upon stimulation with total 15 and 50 mM KCl for all three cell lines (**C**). Mean current densities in baseline (5mM KCl) and upon stimulation with total 15 and 50mM KCl for NCI-H295R (*n* = 51), MUC-1 (*n* = 39) and TVBF-7 (*n* = 26) cells at 80 mV (**D**). Stimulation of the first steroidogenesis steps among the different cell lines and upon different stimulations. CYP11A1 (**E**), HSD3B2 (**F**) and CYP17A1 (**G**) gene expression levels upon FSK stimulation per cell line. Hormonal production of 17-OH progesterone (**H**) in ng per mg of total protein for unstimulated cells in comparison with KCl, AngII, FSK and two different ACTH stimulations. Stars represent significance vs. untreated cells (** *p* < 0.01; *** *p* < 0.001).

Interestingly, comparative characterizations of baseline gene expressions revealed for TVBF-7 much higher levels of Melanocortin 2 Receptor (MC2R) compared to NCI-H295R (NCI-H295R: 100.0 ± 3.9% vs. MUC-1: 0.4 ± 0.1% vs. TVBF-7: 301.4 ± 18.1, *p* < 0.001 for all comparisons), but lower for other hormonal receptors (Angiotensin II receptor type 1-AT1R, Estrogen Receptor 1-ER1, Androgen Receptor-AR and Gonadotropin-Releasing-Hormone Receptor-GNRHR). MUC-1 demonstrate overall rather low or intermediate (Glucocorticoid receptor) baseline expression (Figure 3A). In accordance, also CYP11B1-expression was extraordinary high in TVBF-7 (NCI-H295R: 100.0 ± 3.2%, MUC-1: 0.0 ± 0.0% mV and TVBF-7: 20555.1 ± 991.4%, *p* < 0.001 for all comparisons) and CYP11A1, while for MUC-1 the basal gene expression was found to be overall low or undetectable (Figure 3B). For CYP17A1, HSD3B2, HSD17B4, CYP21A2 and CYP11B2 baseline gene-expression was demonstrated to be highest in NCI-H295R.

Next, the baseline cell sensitivity to potassium (K^+^) was tested electrophysiologically. All cell lines responded to increased concentrations of KCl with depolarization, as shown by the changes in the reversal potential, used as a marker of the resting membrane voltage (Figure 3C, NCI-H295R (*n* = 28–43), MUC-1 (*n* = 25–28) and TVBF-7 (*n* = 24–28)). In the overall presentation for all cell lines, these results are indicated as not-significant for MUC-1, but when the data were analyzed with paired *t*-tests and not as pooled means, the significances were highlighted also in case of MUC-1 (data not shown). Of note, TVBF-7 tend to be the most hyperpolarized (NCI-H295R: −32.11 ± 4.91 mV, MUC-1: −34.74 ± 3.42 mV and TVBF-7: 41.06 ± 4.52, *p* > 0.05 for all comparisons). Considering that, in the presenting data, the liquid junction potential has not been subtracted (calculated around 11 mV for the current setting), the resting membrane voltage of the TVBF-7 cells demonstrates overall the closest value to the known hyperpolarized standard for adrenal cells (around −80 mV). For the same experimental setup, the membrane currents for different KCl concentrations tested at 80 mV remained insignificant for NCI-H295R (*n* = 51), MUC-1 (*n* = 39) and TVBF-7 (*n* = 25) cells (Figure 3D).

### 3.4. Stimulation of the First Steps of Steroidogenesis upon Known Stimuli: TVBF-7 Are Unresponsive

Next, we investigated if the cells respond to known stimulators of steroidogenesis such as KCl, AngII, ACTH and FSK, using FSK as a known inducer of overall steroidogenesis by increasing intracellular cAMP levels. Indeed, FSK stimulation resulted in induction of gene expression of the rate-limiting and first main steps of steroidogenesis (CYP11A1, HSD3B2 and CYP17A1) in NCI-H295R and also MUC-1 cells, while TVBF-7 remained unresponsive (Figure 3E–G). Interestingly, MUC-1 cells responded upon FSK stimulation here even more intensely than NCI-H295R cells. In agreement with these findings, the investigation of 17-OH- Progesterone detected by LC–MS/MS revealed significant increases for NCI-H295R and MUC-1, but not for TVBF-7. Progesterone levels increased for MUC-1 only (NCI-H295R: untreated 0.0 ± 0 vs. FSK 0.0 ± 0 ng/mg protein; MUC-1: untreated 1.6 ± 0.03 vs. FSK 6.8 ± 0.12 ng/mg protein, *p* < 0.001; TVBF-7: untreated 0.12 ± 0.025 vs. FSK 0.13 ± 0.009 ng/mg protein, *p* > 0.999; Supplemental Figure S3).

### 3.5. Mineralocorticoid Pathway Stimulation Reveals Dysregulated and Distinct Patterns among the Cell Lines

**Figure 4:**
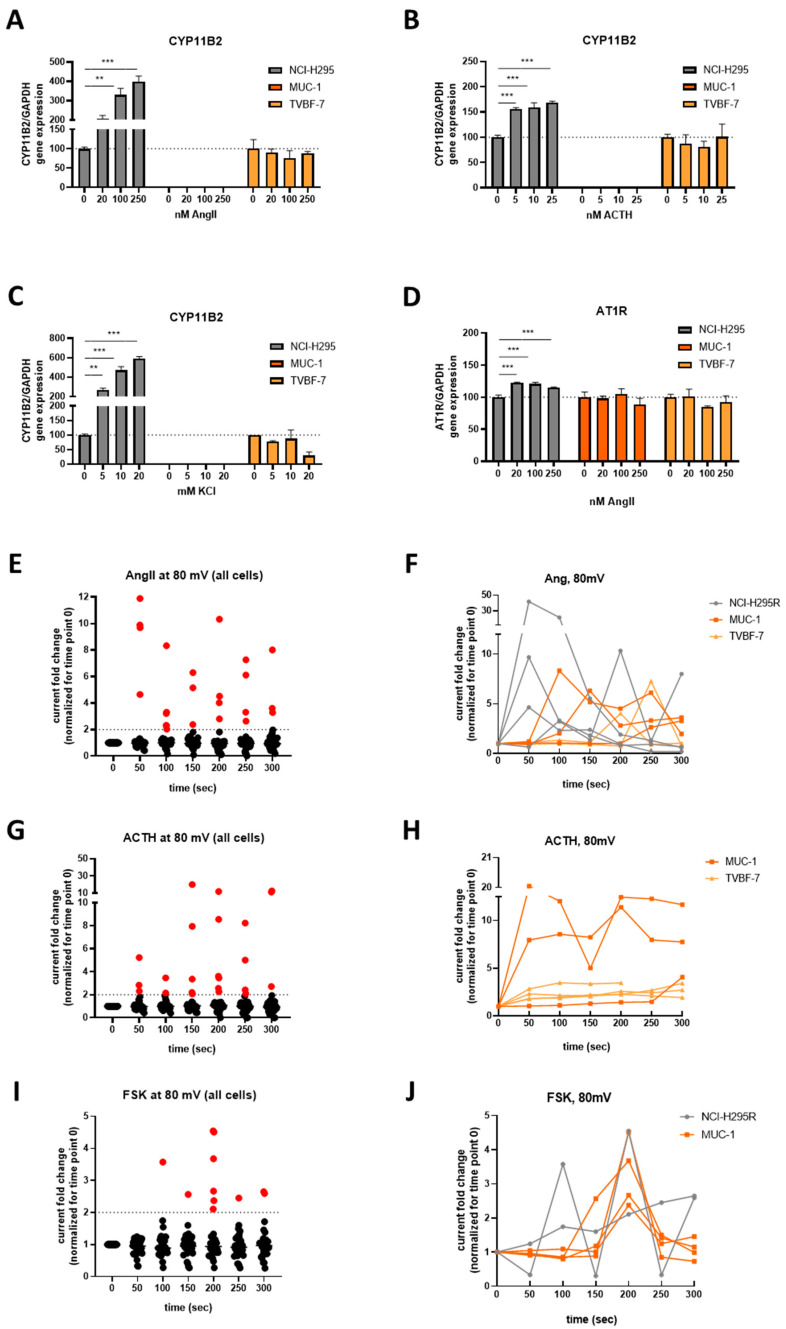
Activation of the mineralocorticoid pathway upon different stimulations. CYP11B2 gene expression upon AngII (**A**), ACTH (**B**) and KCl (**C**) per cell line as well as AT1R expression upon AngII stimulation (**D**). Scatter dot representation of the fold change of the current densities versus baseline at 80 mV for 5 min (300 s) of AngII stimulation cumulatively for all cells per time point aiming to divide the responders (>2 fold change, marked with red) from the non-responders (**E**). Representation of the fold change of the currents of the responder cells in conjunction with time for the AngII stimulation (**F**). Scatter dot representation of the fold change of the current densities versus baseline at 80 mV for 5 min (300 s) of ACTH stimulation cumulatively for all cells per time point aiming to divide the responders (>2 fold change, marked with red) from the non-responders (**G**). Representation of the fold change of the currents of the responder cells in conjunction with time for the ACTH stimulation (**H**). Scatter dot representation of the fold change of the current densities versus baseline at 80 mV for 5 min (300 s) of FSK stimulation cumulatively for all cells per time point aiming to divide the responders (>2 fold change, marked with red) from the non-responders (**I**). Representation of the fold change of the currents of the responder cells in conjunction with time for the FSK stimulation (**J**). Stars represent significance vs. untreated cells (** *p* < 0.01; *** *p* < 0.001).

Next, we have assessed the response of candidates and products of the mineralocorticoid pathway. Hence, stimulation with KCl, AngII and ACTH induced CYP11B2 expression (Figure 4A–C) in NCI-H295R cells but not in TVBF-7 cells while the enzyme expression was undetected in MUC-1. ATR1 gene expression was induced only in NCI-H295R cells upon AngII stimulation (Figure 4D) but not in the other cell lines and not upon KCl or ACTH stimulation (data not shown). The induction towards mineralocorticoid phenotype, though, was not depicted in the hormonal secretion profile within 24 h, since an aldosterone increase was detected for none of the cell lines and 18-OH corticosterone production in NCI-H295R cells upon FSK stimulation only (data are provided in the Supplementary Materials).

AngII stimulation of cells for 5 min (300 s) did furthermore not result in statistically significant changes in reversal potential (Supplemental Figure S4), but regarding membrane currents, various cells responding at different time points and/or consecutive responses of the same cell were detected (change in current density >2 fold of baseline; Figure 4E). Having isolated the responders, we could furthermore observe NCI-H295R (*n* = 3/20) increasing their currents at 80 mV after the first 50 s of stimulation and again, with less intensity in later time points, while MUC-1 (3/16) and TVBF-7 (2/17) responded at 100 s or later (Figure 4F).

### 3.6. Responsive, Non-Producing and Autonomous Secretion Detected Regarding the Glucocorticoid Production

Next, the glucocorticoid production, respective steroidogenic gene and receptor stimulation upon ACTH and FSK stimulation was investigated. While the cells did not show any clear response in terms of reversal potential in the conditions measured (data are in the Supplementary Materials), ACTH stimulation revealed some electrophysiological responders with excessive change in the currents at 80 mV, surprisingly mostly deriving from the MUC-1 (*n* = 3/8) and TVBF-7 (*n* = 4/18) and not from NCI-H295R cells (*n* = 0/15) cells. However, a cell-to-cell analysis did not reveal a specific response pattern (Figure 4G,H). In contrast, upon FSK stimulation, NCI-H295R (*n* = 2/16) and MUC-1 (*n* = 4/15) responded with increases in the current density reaching to a maximum 200 s upon the beginning of FSK stimulation (Figure 4I,J).

**Figure 5:**
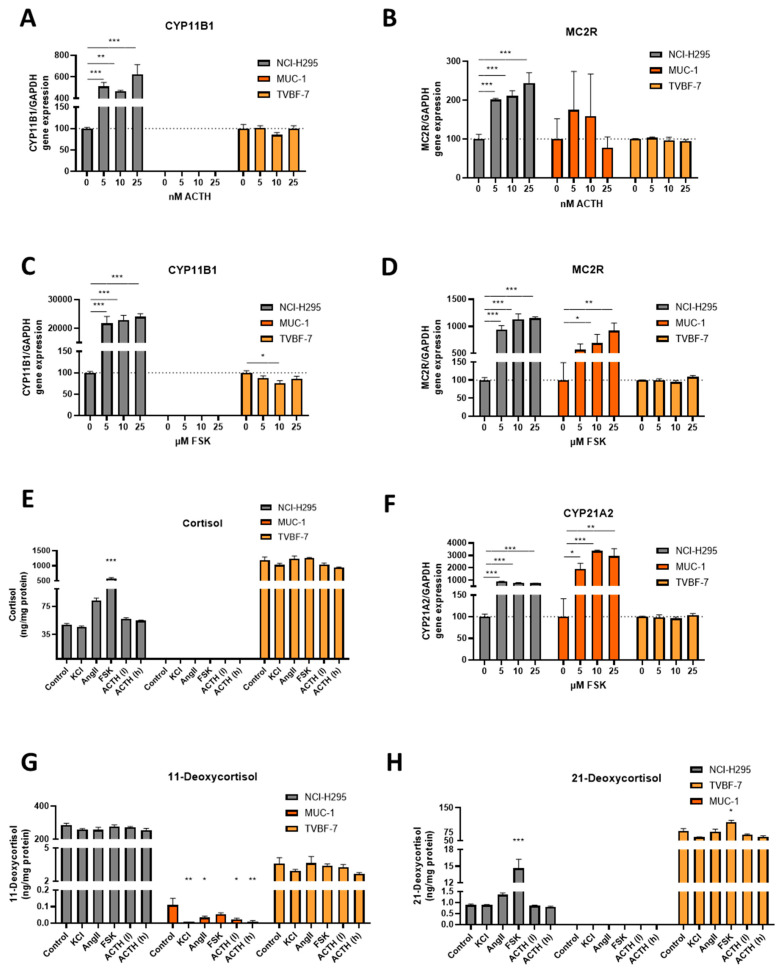
Activation of the glucocorticoid pathway upon different stimulations. CYP11B1 and MC2R gene expression upon ACTH (**A**,**B**) and FSK (**C**,**D**), respectively, per cell line. Hormonal production of cortisol (**E**) in ng per mg of total protein for unstimulated cells in comparison with KCl, AngII, FSK and two different ACTH stimulations. Activation of intermediate steroidogenesis steps upon different stimulations. CYP21A2 gene expression upon FSK stimulation (**F**) per cell line. Hormonal production of 11- (**G**) and 21-deoxycortisol (**H**) in ng per mg of total protein for unstimulated cells in comparison with KCl, AngII, FSK and two different ACTH stimulations. Stars represent significance vs. untreated cells (* *p* < 0.05; ** *p* < 0.01; *** *p* < 0.001).

NCI-H295R responded upon ACTH and NCI-H295R and MUC-1 upon FSK with increase of the MC2R and CYP21A2 gene expression (Figure 5B,D,F) but only in NCI-H295R this was also accompanied by an increase of CYP11B1 gene expression as well as elevation in cortisol and 21-deoxycortisol secretion (Figure 5A,C,E,H). As already indicated before, TVBF-7 demonstrated basally a profile represented by high basal glucocorticoid expression with upregulated MC2R and CYP11B1 expression levels which was again confirmed by extraordinary high basal cortisol (Figure 5E) and 21-Deoxycortisol (Figure 5H) levels. However, in contrast to NCI-H295R they remained unresponsive upon ACTH and FSK stimulation, which corresponds to an autonomous glucocorticoid secretion profile.

### 3.7. MUC-1 Induce Androgen Production and Upregulation of the AR

**Figure 6:**
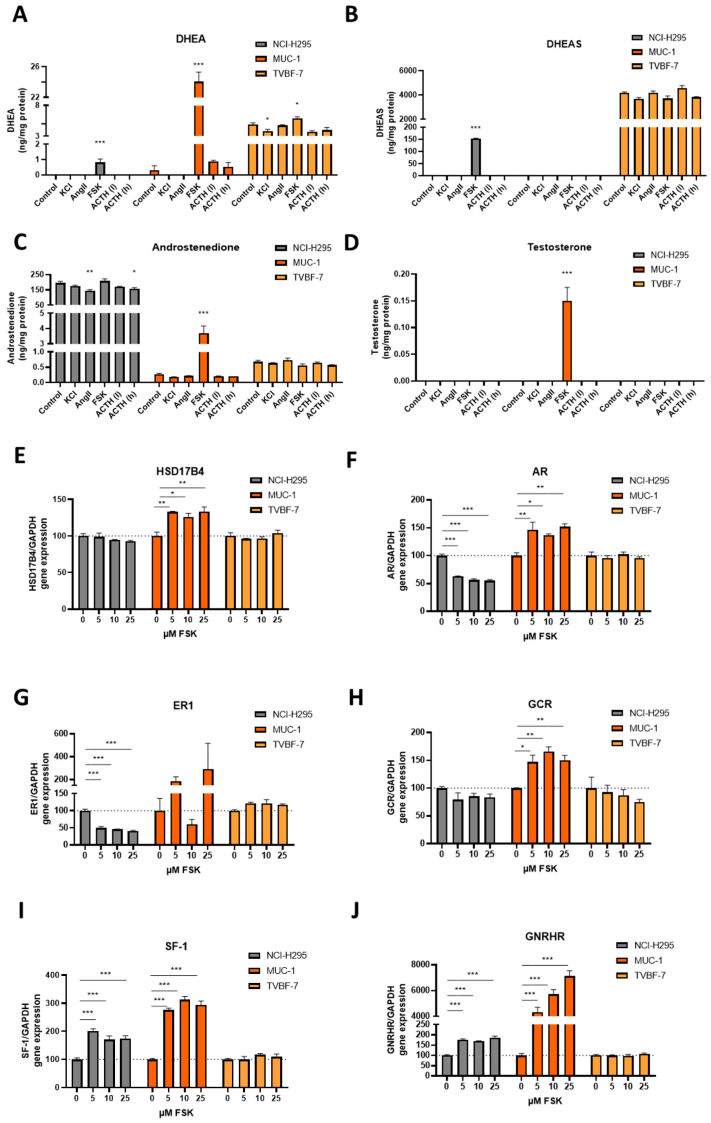
Androgenic pathway activation. Hormonal production of DHEA (**A**), DHEAS (**B**), Androstenedione (**C**) and Testosterone (**D**) in ng per mg of total protein for unstimulated cells in comparison with KCl, AngII, FSK and two different ACTH stimulations. HSD17B4 gene expression upon FSK (**E**) per cell line. Differential response of different hormonal receptor expression per cell 36 line upon FSK stimulation: expression levels of AR (**F**), ER1 (**G**), GCR (**H**), SF-1 (**I**) and GNRHR (**J**) Stars represent significance vs. untreated cells (* *p* < 0.05; ** *p* < 0.01; *** *p* < 0.001).

While NCI-H295R demonstrated basally very high androstendione (Figure 6C) and TVBF-7 DHEAS levels (Figure 6B), only MUC-1 represented a remarkable stimulability and downstream response in the context of androgens (Figure 6A,C,D) which was then by far exceeding DHEA and also Testosterone levels of NCI-H295R and TVBF-7 (Figure 6A,D). Apart from the above-mentioned distinct and around 200 s also specifically patterned electrophysiological response of MUC-1 cells upon FSK stimulation, this was accompanied by highly significant increases in expression of HSD17B4 (Figure 6E), androgen receptor gene (AR, Figure 6F), gonadotropin releasing hormone receptor gene (GNRH, Figure 6J), SF-1 (Figure 6I) and tendencies towards ER1-upregulation (Figure 6G) for MUC-1. Interestingly, in the context of nuclear receptors also the glucocorticoid receptor was under these conditions strongly upregulated for MUC-1 only. In contrast, HSD17B4 remained unchanged, SF-1 was upregulated and AR and ER1 were conversely down-regulated for NCI-H295R. For TVBF-7, again, all levels remained unchanged.

## 4. Discussion

In our study, we have established a novel cell line, namely TVBF-7, which we characterized in direct comparison to the gold standard NCI-H295R and the highly emerging MUC-1 cells. In a first step, we investigated a large panel of known driver genes. Our data demonstrate heterogenous genotypes. For TP53, e.g., we confirmed the known mutations for NCI-H295R and MUC-1 [13] and newly characterized TVBF-7 to be a wildtype. Our studies confirmed also the previously described activating CTNNB1 mutation for NCI-H295R [30]. For TVBF-7, we identified a non-sense APC mutation, while MUC-1 represented wildtype in both genetic loci. Overall, our data suggest a cell panel that includes important tools for preclinical mechanistic and therapeutic studies in the future. The Wnt/β-catenin pathway is a target of many novel treatments suggested for ACC on a preclinical level [31]. Even though a focus of great research, detailed mechanistic and therapeutic studies in a panel of human cell lines naturally reflecting different genotypes are still lacking. Similar considerations can be taken into account for TP53 or other potential candidates. Overall, the initial genomic data revealed that the three cell lines cover important aspects of the mutational heterogeneity met in patients with ACC [32].

In two therapeutic studies, NCI-H295R, MUC-1 and TVBF-7 (as primary culture ACC115m) have been already implemented. Interestingly, independent from genetic status both metastatic models often shared rather less therapeutic responsiveness compared to NCI-H295R [27,29], a therapeutic phenotype which is already well known from further studies implementing MUC-1 [20,21,22,23,25,26,28]. It should be mentioned in this context that, in contrast to the primary derived chemo-naive NCI-H295R, both metastatic models were obtained from EDP-M treated patients [15,27]. However, patients with advanced, metastatic and pre-treated disease are the ones urgently requiring ongoing multi-chemotherapeutic treatments, while early stages with a low risk of recurrence undergo in first-instance surgery [1]. Thus, in the best case, preclinical platforms provide such patient tumor heterogeneities as various stages, therapeutic responsiveness, genotypes, hormonal phenotypes and gender, among others.

Consequently, we went on with the investigation of other important aspects such as steroidogenic signaling, secretion and, electrophysiological responsiveness using an innovative and unbiased automated high-through-put patch-clamp system. Alongside maintenance in characteristics of steroid producing cells such as the most hyperpolarized reversal potential and sensitivity to increased potassium concentrations [33], our studies revealed for TVBF-7 extremely high expression of MC2R, CYP11B1, cortisol and DHEAS secretion at baseline. The cells were furthermore unresponsive to a selection of known physiological stimuli. A profile comparable to autonomous cortisol secretion could be found [34]. Hypercortisolism in such patients is often mild, and most patients lack typical clinical features of overt Cushing’s syndrome. Likewise, TVBF-7 was derived from a patient without obvious clinical signs of hormone excess [27]. However, a correlation of APC inactivating mutations and elevated cortisol levels has been already previously reported for patients with adrenal tumors [30,35].

In contrast, for MUC-1 which was also derived from a male patient tumor originally diagnosed as hormonally diffuse with no clinical signs of steroid excess [15,36] at baseline no parameter of steroid excess was detectable. Fittingly, MUC-1 demonstrated a profile with comparably low steroidogenic activity and secretion. However, upon FSK stimulation MUC-1 demonstrated the capacity to induce impressively strong main steroidogenic genes such as CYP11A1, HSD3B2, CYP17A1 and also the upregulation of genes involved in glucocorticoid metabolism such as MC2R and CYP21A2. Electrophysiologically, MUC-1 responded furthermore to FSK showing a clear pattern, which is in alignment with the respective outcome for gene expression and hormonal levels. Interestingly, in previous studies with MUC-1 xenografts and first passages after explantation from a female host, MUC-1 demonstrated increased cortisol levels [15]. Hormonal patterns in ACC might be, thus, conditional and inducible by a variety of local factors. Tumors, clinically considered as not functional, might be thereby still able to lead to profound hormonal changes within the tumor-microenviroment, even if not leading to whole body excess. This finding could be of high clinical relevance in the context of therapeutic treatments and responsiveness. Moreover, also gender aspects could be involved. Data which are scarce in the literature concerning the effects of gender on adrenal tumors [37]. However, it is known that cortisol-secreting adrenal tumors are more often diagnosed in female patients [38]. Of note, SF-1 is a key regulator of human sex determination [39] and its activation might lead to different downstream effects in tissues of male and female origin.

Androgens, estrogens, and progestins are known as sex steroids. For prostate and breast cancer mechanisms of sex-steroid hormone–regulated DNA damage repair is already well known [40]. Recently, our group reported marked differences in DNA damage repair leading to a rather drug-resistant phenotype for MUC-1 vs. NCI-H295R [20]. Together with the data obtained from the current study, it is prudent to speculate about a potential involvement of AR and its ligands in the drug resistant phenotype of MUC-1. Of note, the AR was markedly differently regulated in NCI-H295R (down), MUC-1 (up) and TVBF-7 (unchanged). The main AR ligand testosterone was within the observed time-frame detectable for MUC-1 exclusively, as well as an extraordinary increase in DHEA. DHEA is known to augment AR levels, and, furthermore, is able to compete with dihydrotestosterone indicating intrinsic androgenic activity that is potentially independent of metabolic conversion to other androgens and can affect gene function through the AR [41]. In contrast, further studies demonstrate that androgenicity of DHEAS, which is instead rather elevated in NCI-H295R and even more in TVBF-7, is negligible [42]. However, also other nuclear receptors (ER and GCR) underly differential regulation in the various models. In this context, it is relevant to mention that previous studies demonstrated at baseline higher HSP90-abundance in MUC-1 compared to NCI-H295R, which was again correlated with less therapeutic responsiveness towards therapeutic HSP90 inhibition for MUC-1 [22]. Overall, further studies will be required, to elucidate the underlying mechanisms of action under varying conditions in more detail.

Interestingly, also HSD17B4 a gene with a dual role in steroidogenesis and fatty acid oxidation is specifically upregulated for MUC-1 under FSK-conditions. HSD17B4 expression is known to increase in castration resistant prostate cancer, leading to metabolic re-programming resulting in AR-stimulation and poor prognosis [43]. Furthermore, a recent study reported a main role in the context of mitochondrial and peroxisomal fatty acid oxidation and that the latter one can serve as a compensatory mechanism in case of mitochondrial defects or overload [44]. This is in the specific context of great interest, as we have recently reported significant differences in cholesterol storage between mitotane sensitive (NCI-H295R) and resistant (MUC-1) adrenocortical cells [21]. Furthermore, upcoming data indicate strong differences in storage of cholesterylester-containing lipid droplets (NCI-H295R) vs. triacyglycerol-LDs (MUC-1) and the appropriate differential lipid-metabolism in this context [45].

In sum, the aim of the current study was to characterize NCI-H295R, MUC-1 and TVBF-7 regarding their underlying heterogenic geno- and phenotypes, to thereby reveal varying steroidogenic and down-stream signalling/secretion capacities and highlight their significant, but not interchangeable values for new therapeutic and mechanistic studies by representing various clusters known for ACC patients.

## Data Availability

All data that were needed to evaluate the conclusions in the paper are present in the paper and/or the Supplementary Materials. The cell lines MUC-1 and TVBF-7 can be provided by C. Hantel and S. Sigala/A. Berruti, respectively, pending scientific review and completed MTAs.

## Supplementary Materials

The following supporting information can be downloaded at: https://www.mdpi.com/2073-4409/11/9/1439/s1, Figure S1: AT1R gene expression upon increasing KCl (**A**) and ACTH stimulation (**B**) and MC2R gene expression upon increasing KCl (**C**) and AngII stimulation (**D**). Stars represent significance vs. untreated cells (*, *p* < 0.05; **, *p* < 0.01; ***, *p* < 0.001); Figure S2: Hormonal production of Aldosterone (**A**), 18-oxo-cortisol (**B**), 18-OH-cortisol (**C**) and 11-deoxycorticosterone (**D**) in ng per mg of total protein for unstimulated cells in comparison with KCl, AngII, FSK and two different ACTH stimulations. Stars represent significance vs. untreated cells (*, *p* < 0.05; **, *p* < 0.01; ***, *p* < 0.001); Figure S3: Hormonal production of 18-OH-corticosterone (**A**), Pregnenolone (**B**), Progesterone (**C**) and cortison (**D**) in ng per mg of total protein for unstimulated cells in comparison with KCl, AngII, FSK and two different ACTH stimulations. Stars represent significance vs. untreated cells (*, *p* < 0.05; **, *p* < 0.01; ***, *p* < 0.001); Figure S4: Reversal potential changes over time upon stimulation with AngII (**A**), ACTH (**B**) and Forskolin (FSK) (**C**) for all three cell lines. Table S1: STR Profile at the University of Brescia; Table S2: Primers used in the Quantitative Real-Time PCR; Table S3: Single-nucleotide polymorphism (SNP) findings for certain ACC and general cancer genes filtered for mutations located in coding (exonic) regions, with exclusion of the synonymous (silence) mutations.
