# Correction: Sigala et al. A Comprehensive Investigation of Steroidogenic Signaling in Classical and New Experimental Cell Models of Adrenocortical Carcinoma. *Cells* 2022, *11*, 1439

**DOI:** 10.3390/cells12182274

**Published:** 2023-09-14

**Authors:** Sandra Sigala, Christina Bothou, David Penton, Andrea Abate, Mirko Peitzsch, Deborah Cosentini, Guido A. M. Tiberio, Stefan R. Bornstein, Alfredo Berruti, Constanze Hantel

**Affiliations:** 1 Section of Pharmacology, Department of Molecular and Translational Medicine, University of Brescia, 25124 Brescia, Italy; sandra.sigala@unibs.it (S.S.); a.abate005@unibs.it (A.A.); 2 Department of Endocrinology, Diabetology and Clinical Nutrition, University Hospital Zurich (USZ) and University of Zurich (UZH), 8091 Zürich, Switzerland; christina.bothou@usz.ch (C.B.); stefan.bornstein@uniklinikum-dresden.de (S.R.B.); 3 Electrophysiology Facility (e-phac), Department of Molecular Life Sciences, University of Zurich (UZH), 8057 Zürich, Switzerland; david.pentonribas@uzh.ch; 4 Medizinische Klinik und Poliklinik III, University Hospital Carl Gustav Carus Dresden, 01307 Dresden, Germany; mirko.peitzsch@uniklinikum-dresden.de; 5 Medical Oncology Unit, Department of Medical and Surgical Specialties, Radiological Sciences, and Public Health, University of Brescia at ASST Spedali Civili di Brescia, 25124 Brescia, Italy; deborah.cosentini@gmail.com (D.C.); alfredo.berruti@unibs.it (A.B.); 6 Surgical Clinic, Department of Clinical and Experimental Sciences, University of Brescia at ASST Spedali Civili di Brescia, 25124 Brescia, Italy; guido.tiberio@unibs.it; 7 Diabetes and Nutritional Sciences, King’s College London, London WC2R 2LS, UK; 8 Center for Regenerative Therapies, Technische Universität Dresden, 01307 Dresden, Germany; 9 Paul-Langerhans-Institute Dresden, Helmholtz Center Munich, University Hospital Carl Gustav Carus, Faculty of Medicine, Technische Universität Dresden, 01307 Dresden, Germany; 10 Lee Kong Chian School of Medicine, Nanyang Technological University, Singapore 636921, Singapore

## Error in Figure

The authors made the following changes to their paper [1]. The authors noticed a mistake on the SF-1 results for the cell line NCI-H295 in Figure 6. A mistake happened during data transfer for graph preparation. The authors are very sorry about this error. They have now repeated the specific experiment for a total of five times; it was performed by two independent persons and with two different primer pairs (only the original primer pair is included here, but two different primer pairs were used to ensure that the effect between different SF-1 primers is consistent).

Figure 6I (marked in red) was corrected from


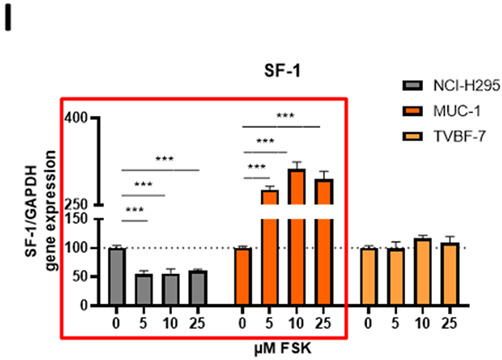


to


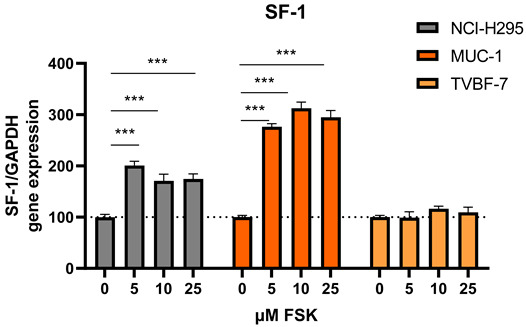


## Text Correction

A correction has been made to Section 3.7:

The sentence “In contrast, HSD17B4 remained unchanged and the AR, ER1 and SF-1 were conversely down-regulated for NCI-H295R.For TVBF-7, again, all levels remained unchanged” was modified to “In contrast, HSD17B4 remained unchanged, SF-1 was upregulated and AR and ER1 were conversely down-regulated for NCI-H295R. For TVBF-7, again, all levels remained unchanged”.

A correction has been made to Section 4, paragraphs 4 and 5:

The sentence “Of note, SF-1 is a key regulator of human sex determination [39] and was upon FSK stimulation strikingly different regulated in NCI-H295R (down), MUC-1 (up) and TVBF-7 (unchanged)” was corrected to “Of note, SF-1 is a key regulator of human sex determination [39] and its activation might lead to different downstream effects in tissues of male and female origin”.

The sentence “Of note, the AR was again markedly different regulated following the same patterns as observed for SF-1 in NCI-H295R (down), MUC-1 (up) and TVBF-7 (unchanged)” was corrected to “Of note, the AR was markedly differently regulated in NCI-H295R (down), MUC-1 (up) and TVBF-7 (unchanged)”.

The authors state that the scientific conclusions are unaffected. This correction was approved by the Academic Editor. The original publication has also been updated.
