# Supplementary material for: A Comprehensive Investigation of Steroidogenic Signaling in Classical and New Experimental Cell Models of Adrenocortical Carcinoma
Source: Cells. 2022 Apr 24;11(9):1439. doi: 10.3390/cells11091439 (PMC9103477; doi:10.3390/cells11091439)

Supplementary Materials:

A. Supplementary materials and methods:

Whole genome sequencing and bioinformatics analysis:

Whole genome sequencing (WGS) for NCI-H295R, MUC-1 and TVBF-7 cells has been performed and standard bioinformatic analysis has been carried out (BGI, Shenzhen, Guangdong, China). More specifically, for the bioinformatics analysis, data were initially filtered for removing adaptors, contamination, and low-quality reads from raw reads and consecutively the reads were aligned to the human reference genome (UCSC build HG19) using Burrows-Wheeler Aligner (BWA) software. Moreover, assessment of sequencing quality, including data production statistics, sequencing depth distribution and coverage uniformity was performed and then variant calling, BQSR (Base Quality Score Recalibration) and GVCF calling (tools: GATK). Following, Single-nucleotide polymorphism (SNP) calling (tools: GATK), SNP VQSR (Variant Quality Score Recalibration), SNP VQSR Filtering and SNP annotation (annotations of each SNP to the corresponding gene functional units in RefGene database, including nucleotide and amino acid changes, etc.), SNP validation and comparison (with dbSNP database, 1000 Genomes Project database, ESP) and the respective statistics took place. Finally, a functionality and conservation prediction of SNPs took place and the whole procedure as in SNPs was repeated for insertion and deletion (InDel) and copy number variations (CNVs) have been performed. The analysis data were annotated with the use of SnpEff tool (http://snpeff.sourceforge.net/SnpEff_manual.html) and the VEP tool (https://asia.ensembl.org/info/docs/tools/vep/index.html), the data were delivered post-analysis in vcf files which were further assessed by the use of EmEditor (Washington, US) for the generation of the information provided in Figure 2B and in the Supplementary Table 3.

Supplementary Table S1: STR Profile at the University of Brescia

TVBF-7 STR Profiling	
**Marker**	**519631 - D1**
AMEL	X
D3S1358	17
D1S1656	16
D2S441	12
D10S1248	14
D13S317	12
Penta E	5
D16S539	8
D18S51	19
D2S1338	19
CSF1PO	12
Penta D	9 12
TH01	6
vWA	17
D21S11	29
D7S820	11 12
D5S818	12
TPOX	8
DYS391	--
D8S1179	15
D12S391	18 20
D19S433	13
FGA	21
D22S1045	15

Supplementary Table S2: Primers used in the Quantitative Real- Time PCR.

Name	Forward Primer (5'-3')	Reverse Primer (5'-3')	Product size (bp)	Company
Cyp11b1	TCCCGAGGGCCTCTAGGA	GGGACAAGGTCAGCAAGA	85	Microsynth (Balgach, Switzerland)
Cyp11a1	GAGATGGCACGCAACCTGAAG	CTTAGTGTCTCCTTGATGCTGGC	137	Microsynth
Cyp17a1	TGGCCCCATCTATTCGGTTC	CTTCTCCAGCTTCTGATCG	253	Microsynth
Cyp21a2	AGAGGGATCACATCGTGGAG	CTTCCAGGAGCTGTCCAG	141	Microsynth
HSD3B2	CTAAGTTACGCCCTCTTCTG	AATGTCTCCTTCAAGTACA	289	Microsynth
Cyp11b2	AGCTGGGACATTGGTACAGGT	GCATGCCAAAGCCAAAGG	149	Microsynth

MC2R	CATGGGCTATCTCAAG CCAC	GAGATCTTCCTGGTGTGG	360	Microsynth
AT1R	CAGATGACGGCTGCTC GAAG	TGGAAACTGGACAGAACA	200	Microsynth
HSD17B4	TGAGGGATCGTTCCTTT GCTA	CGTGTCACTTGGAATGAA	88	Microsynth
Ki-67	TCCTTTGGTGGGCACCT AAGACCTG	TGATGGTTGAGGCTGTTCC	155	Microsynth
GAPDH	AGCCTCCCGCTTCGCTC TCT	CCAGGCGCCCAATACGAC	163	Microsynth
hSF1	CAGCCTGGATTTGAAG TTCCT	CAGCATTTCGATGAGCAG	237	Microsynth
AR	CGGAAGCTGAAGAAAC TTGG	ATGGCTTCCAGGACATTC	155	#NM_000044, RealTimePrimers, Melrose Park, PA, USA
GNRHR	ATCAACAACAGCATCC CACT	GTCGCAGAGAGCAGAAAA	104	#NM_000406, RealTimePrimers
ER 1	GAGGATTCCCGTAGCT CTTC	CCCTTGACCTAGCTTTCTC C	211	#NM_000125, RealTimePrimers
GCR	GGTGCTGTTTGAAAGC AGAT	CCAAGGTTTCCTCCCATAG	240	# NM_001018077, RealTimePrimers

B. **Supplementary Results:**

C. **Supplementary Table S3:** Single-nucleotide polymorphism (SNP) findings for certain ACC and general cancer genes filtered for mutations located in coding (exonic) regions, with exclusion of the synonymous (silence) mutations.

Gene Name	Cell line	Type of alteration	Mutation	SNP Database ID
TP53	*MUC-1, TVBF-7*	missense	NM_000546: c.C215G (p.P72R)	rs1042522
MEN1	*NCI-H295R, MUC-1, TVBF-7*	missense	NM_000244: c.A1636G (p.T546A)	rs2959656
	MUC-1	missense	NM_000244: c.G527A (p.R176Q)	rs607969
PRKAR1A	*NCI-H295R*	missense	NM_001276290: c.G998A (p.S333N)	rs9789047
CTNNB1	*No finding*			
APC	*NCI-H295R, TVBF-7*	missense	NM_000038: c.T5465A (p.V1822D)	rs459552
ZNRF3	*No finding*			
INS-IGF2	*MUC-1, TVBF-7*	missense	NM_001042376: c.T431C (p.L144P)	rs10770125
EGFR	*NCI-H295R*	missense	NM_201284: c.C2108T (p.S703F)	rs10258568
	TVBF-7	missense	NM_005228.5: c.G1562A (p.R521K)	rs2227983
RB1	*No finding*			
BRCA1	*MUC-1*	missense	NM_007297: c.A926G (p.Q309R)	rs1799950
	NCI-H295R	missense	NM_007297: c.A4696G (p.S1566G)	rs1799966
	NCI-H295R	missense	NM_007297: c.A3407G (p.K1136R)	rs16942
	NCI-H295R	missense	NM_007297: c.C2471T (p.P824L)	rs799917
	NCI-H295R	missense	NM_007297: c.A2317G (p.K773E)	rs56082113
BRCA2	*NCI-H295R, MUC-1,*	missense	NM_000059: c.T7397C (p.V2466A)	rs169547
	MUC-1	missense	NM_000059: c.A865C (p.N289H)	rs766173
	MUC-1	missense	NM_000059: c.A2971G (p.N991D)	rs1799944
	TVBF-7	missense	NM_000059: c.A1114C (p.N372N)	rs144848
RET	*NCI-H295R, MUC-1*	missense	NM_020630: c.G2071A (p.G691S)	rs1799939
GNAS	*NCI-H295R*	missense	NM_001077490: c.C940T (p.R314W)	rs7121
PTEN	*NCI-H295R, MUC-1, TVBF-7*	missense	NM_001304717: c.G194C (p.C65S)	rs2943772

Supplementary Figure S1: AT1R gene expression upon increasing KCl **(A)** and ACTH stimulation **(B)** and MC2R gene expression upon increasing KCl **(C)** and AngII stimulation **(D)**. Stars represent significance vs. untreated cells (*, $p < 0.05$; **, $p < 0.01$; ***, $p < 0.001$).

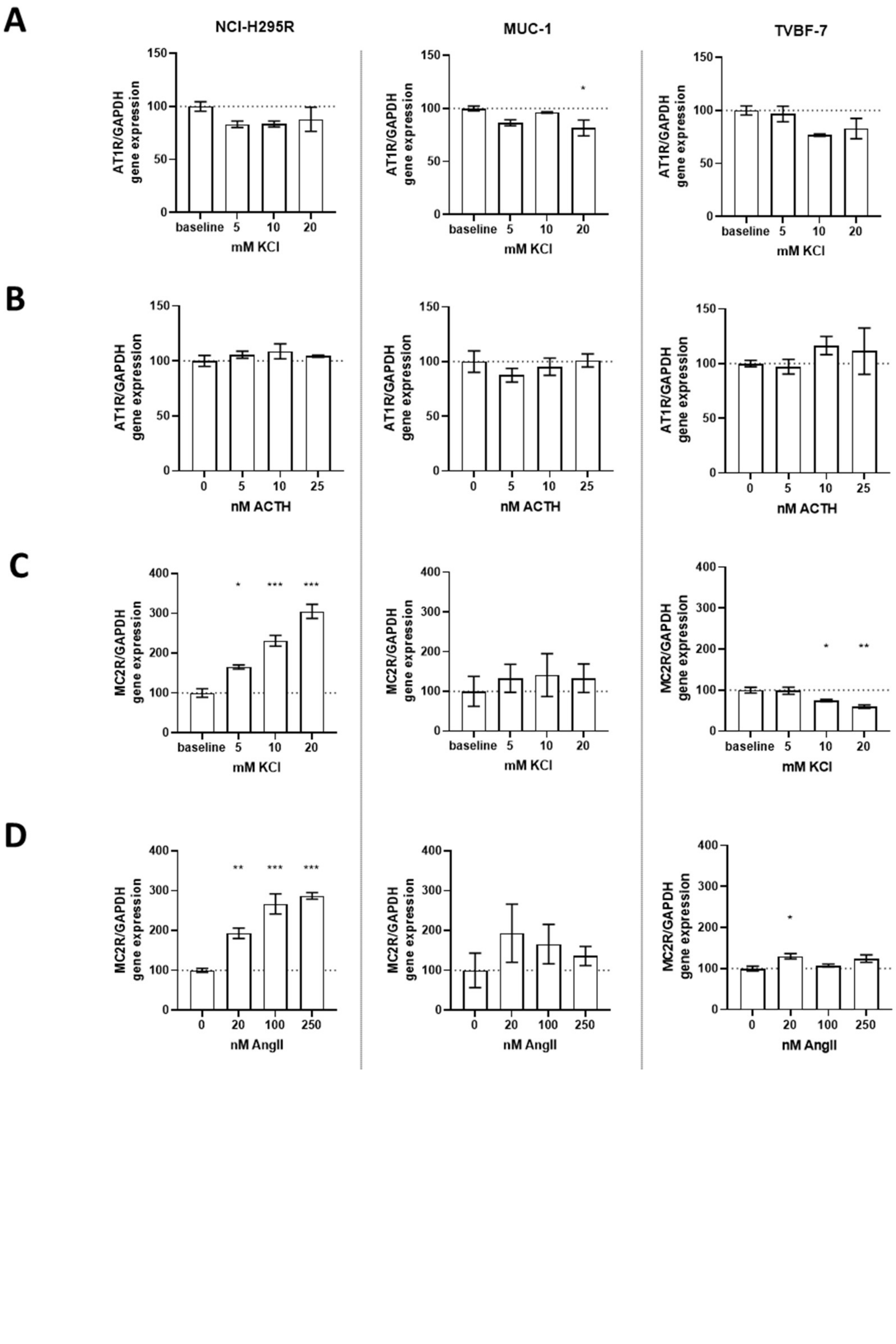

Supplementary Figure S2: Hormonal production of Aldosterone **(A)**, 18-oxo-cortisol **(B)**, 18-OH-cortisol **(C)** and 11-deoxycorticosterone **(D)** in ng per mg of total protein for unstimulated cells in comparison with KCl, AngII, FSK and two different ACTH stimulations**.** Stars represent significance vs. untreated cells (*, $p < 0.05$; ***, $p < 0.001$).

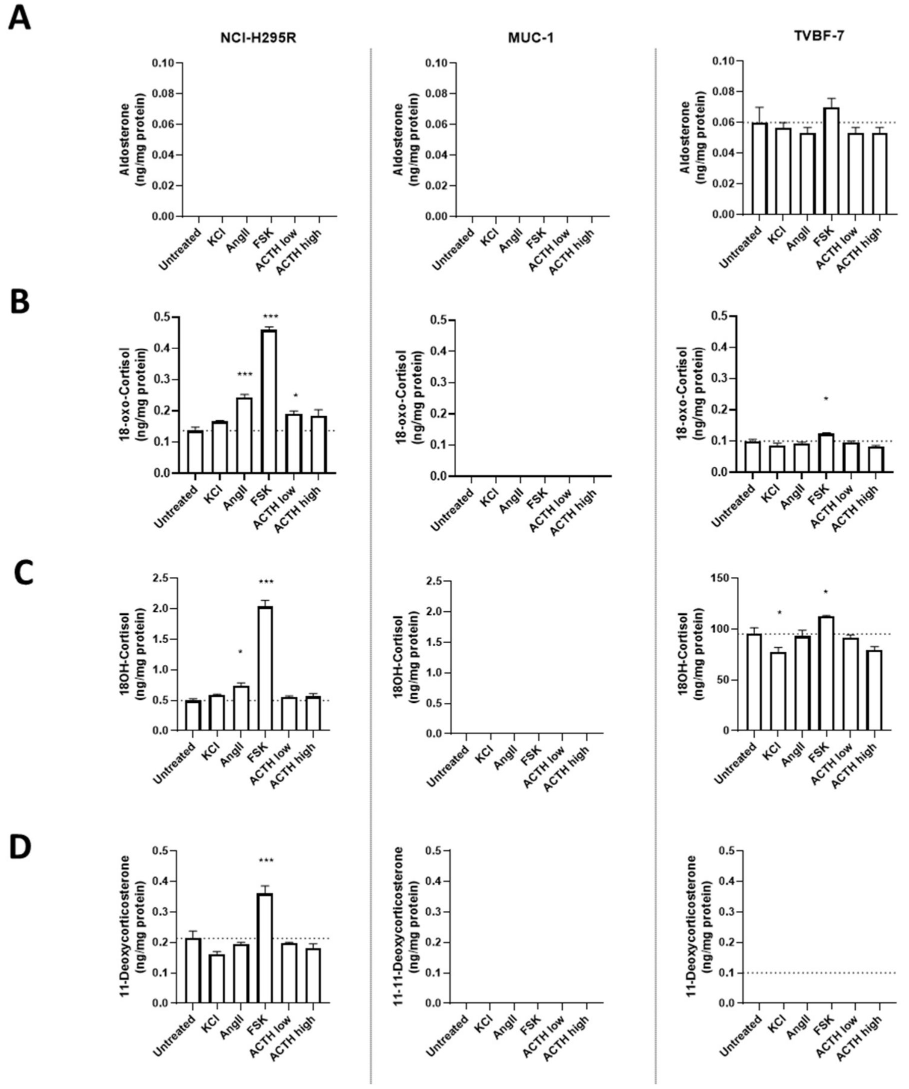

Supplementary Figure S3: Hormonal production of 18-OH-corticosterone **(A)**, Pregnenolone **(B)**, Progesterone **(C)** and cortison **(D)** in ng per mg of total protein for unstimulated cells in comparison with KCl, AngII, FSK and two different ACTH stimulations**.** Stars represent significance vs. untreated cells (*, $p < 0.05$; **, $p < 0.01$; ***, $p < 0.001$).

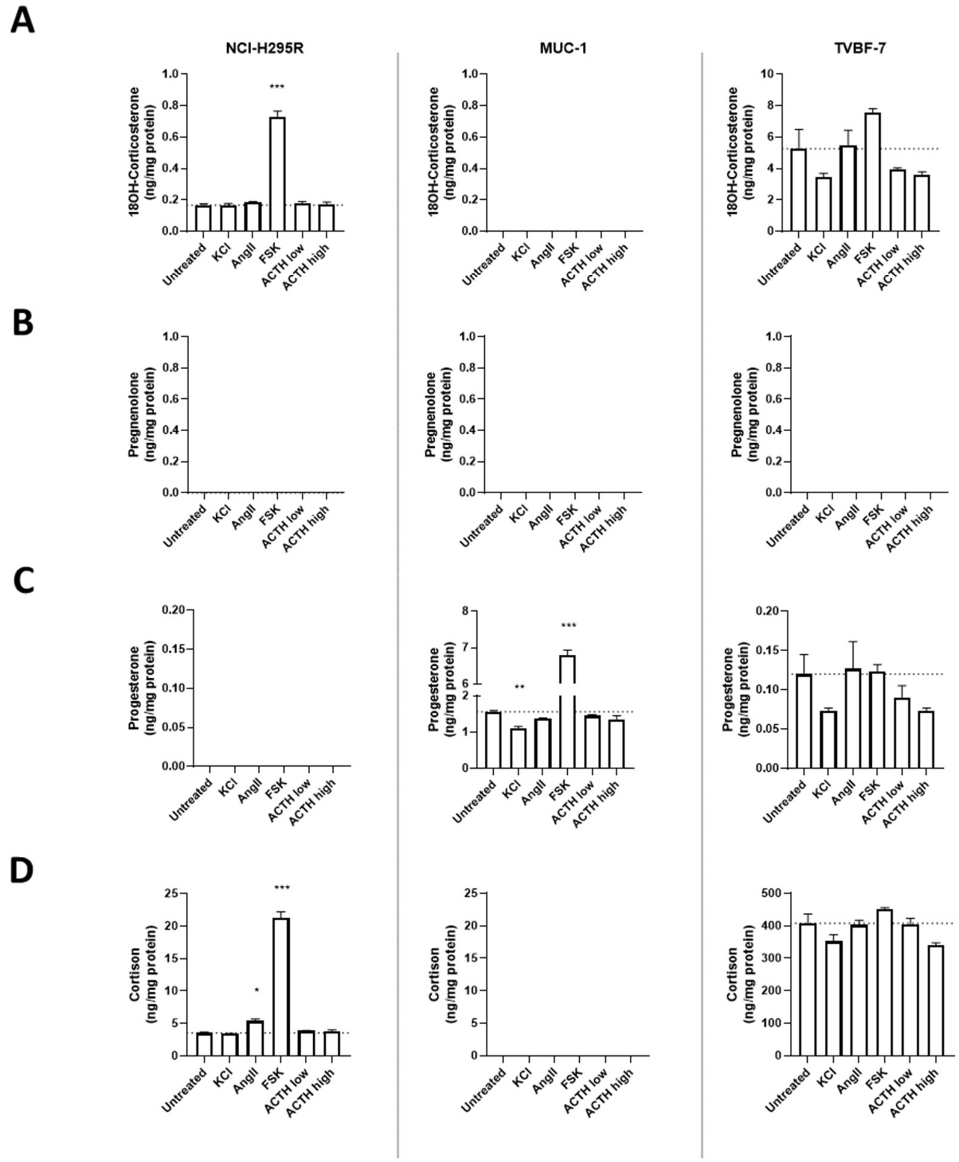

Supplementary Figure S4: Reversal potential changes over time upon stimulation with AngII **(A)**, ACTH **(B)** and Forskolin (FSK) **(C)** for all three cell lines.

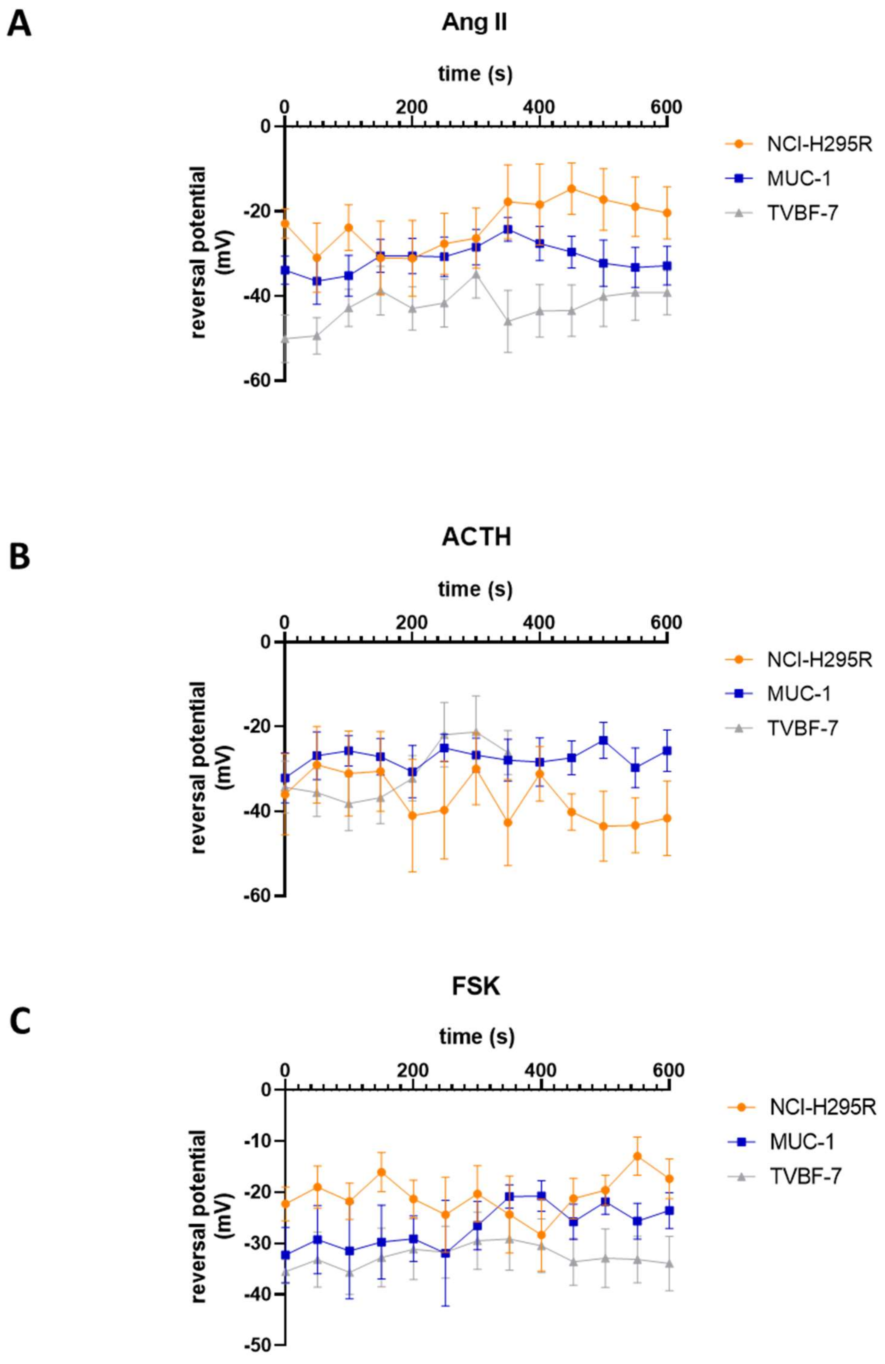

Supplement: Supplementary file 1 [file cells-11-01439-s001.zip › cells-1658167-supplementary.pdf]
